# Little Evidence of Avian or Equine Influenza Virus Infection among a Cohort of Mongolian Adults with Animal Exposures, 2010–2011

**DOI:** 10.1371/journal.pone.0085616

**Published:** 2014-01-21

**Authors:** Nyamdavaa Khurelbaatar, Whitney S. Krueger, Gary L. Heil, Badarchiin Darmaa, Daramragchaa Ulziimaa, Damdindorj Tserennorov, Ariungerel Baterdene, Benjamin D. Anderson, Gregory C. Gray

**Affiliations:** 1 Mongolian Association for Infectious Diseases Researchers, Ulaanbaatar, Mongolia; 2 College of Public Health and Health Professions, and Emerging Pathogens Institute, University of Florida, Gainesville, Florida, United States of America; 3 National Influenza Center, National Center of Communicable Diseases, Ministry of Health, Ulaanbaatar, Mongolia; 4 National Center for Zoonotic Diseases, Ministry of Health, Ulaanbaatar, Mongolia; Thomas Jefferson University, United States of America

## Abstract

Avian (AIV) and equine influenza virus (EIV) have been repeatedly shown to circulate among Mongolia’s migrating birds or domestic horses. In 2009, 439 Mongolian adults, many with occupational exposure to animals, were enrolled in a prospective cohort study of zoonotic influenza transmission. Sera were drawn upon enrollment and again at 12 and 24 months. Participants were contacted monthly for 24 months and queried regarding episodes of acute influenza-like illnesses (ILI). Cohort members confirmed to have acute influenza A infections, permitted respiratory swab collections which were studied with rRT-PCR for influenza A. Serologic assays were performed against equine, avian, and human influenza viruses. Over the 2 yrs of follow-up, 100 ILI investigations in the cohort were conducted. Thirty-six ILI cases (36%) were identified as influenza A infections by rRT-PCR; none yielded evidence for AIV or EIV. Serological examination of 12 mo and 24 mo annual sera revealed 37 participants had detectable antibody titers (≥1∶10) against studied viruses during the course of study follow-up: 21 against A/Equine/Mongolia/01/2008(H3N8); 4 against an avian A/Teal/Hong Kong/w3129(H6N1), 11 against an avian-like A/Hong Kong/1073/1999(H9N2), and 1 against an avian A/Migrating duck/Hong Kong/MPD268/2007(H10N4) virus. However, all such titers were <1∶80 and none were statistically associated with avian or horse exposures. A number of subjects had evidence of seroconversion to zoonotic viruses, but the 4-fold titer changes were again not associated with avian or horse exposures. As elevated antibodies against seasonal influenza viruses were high during the study period, it seems likely that cross-reacting antibodies against seasonal human influenza viruses were a cause of the low-level seroreactivity against AIV or EIV. Despite the presence of AIV and EIV circulating among wild birds and horses in Mongolia, there was little evidence of AIV or EIV infection in this prospective study of Mongolians with animal exposures.

## Introduction

Among the world’s last pastoral people groups, Mongolians often live in close proximity with flocks of migrating birds or free-ranging herds of horses. Mongolia’s large migrating bird populations have been shown to harbor both highly-pathogenic and low-pathogenic avian influenza viruses (AIV) [1], [2], [3], [4]. In addition, having some of the highest horse-to-man population ratios in the world, Mongolia has suffered some of the world’s largest equine influenza A virus (EIV) epizootics [5]. In recent years H3N8 EIV epizootics occurred in 2007–2008 (96,390 cases; 24,600 deaths) and again in 2011 (75,208 cases; 40 deaths) (Mongolia’s Department of Veterinary and Animal Breeding). In discussions with rural Mongolians, we learned that when their horses became sick, their children sometimes suffered upper respiratory tract infections with similar symptoms. Knowing humans can experience AIV infections and H3N8 EIV has been experimentally shown to infect volunteers who were intranasally inoculated [6], we sought to prospectively study Mongolians for evidence of AIV and EIV infections.

## Materials and Methods

### Ethics Statement

This study was approved by IRBs at the University of Florida, the National Center of Communicable Diseases, Mongolia, and the US Army Medical Department. Written informed consent was obtained from each participant.

### Study Design

Details about the study location, study subjects, enrollment methods, database generation, and serology laboratory methods have previously been published [7]. Briefly, Mongolians greater than 18 yrs of age were recruited from 3 regions in Mongolia and followed with monthly encounters over a 24-month period for evidence of influenza-like-illness. Sera and questionnaire data were collected at enrollment, 12 months, and 24 months. Annual follow-up questionnaires collected demographic, health, and animal exposures data during the past year. Poultry or horse exposure was defined as contact ≥5 cumulative hour/week for at least one week.

### Monthly Follow-up

During enrollment, cohort participants were given oral and written instructions and a digital thermometer. They were asked to contact study field staff upon developing signs and symptoms of an influenza-like illness (ILI) via a telephone call. Study staff also conducted monthly home visits to remind participants of the importance of reporting ILI and to assess whether an illness was present or had occurred during the preceding week. ILI was defined as acute onset of a respiratory illness with an oral (or equivalent from other body region) measured temperature ≥38°C and a sore throat, cough, shortness of breath, or respiratory distress for 4 or more hours.

### Investigating Influenza-like Illness

When a possible ILI was reported to study staff, a home visit was performed within 24 hrs of notification. If the subject met the ILI case definition, a study nurse completed an ILI questionnaire and collected 2 respiratory swab specimens (nasal and pharyngeal). The swab specimens were stored in viral transport media and transported using cold-chain within 24 hrs after collection to local field laboratories in Khovd and Dornogovi provinces and to the National Influenza Center in Ulaanbaatar.

### Laboratory Methods

Sera and ILI respiratory swab aliquots were preserved at −80°C and transported on dry ice to the University of Florida for testing. Sera were tested for evidence of human, equine, and avian influenza infections over time (Table 1). ILI swabs were studied for molecular evidence of influenza A virus RNA.

**Table 1:** Viruses used in serological studies.

Avian viruses	Human viruses
A/Migratory duck/Hong Kong MPS180/2003(H4N6)	A/Brisbane/59/2007(H1N1)*
A/Cygnus/Mongolia/3/2009(H5N1)†	A/Mexico/4108/2009(H1N1)* ‡
A/Nopi/Minnesota/2007/462960-2(H5N2)	A/Brisbane/10/2007(H3N2)*
A/Teal/Hong Kong/w312/97(H6N1)	
A/Water fowl/Hong Kong/Mpb127/2005(H7N7)	**Equine virus**
A/Migratory duck/Hong Kong/MP2553/2004(H8N4)	A/Equine/Mongolia/01/2008(H3N8)
A/Hong Kong/1073/1999(H9N2)**	
A/Migratory duck/Hong Kong/MPD268/2007(H10N4)	
A/Chicken/New Jersey/15906-9/1996(H11N1)	
A/Duck/Alberta/60/1976(H12N5)	

Unless otherwise indicated, serologic study was performed using the microneutralization assay.

Virus studied with hemagglutination inhibition assay

Highly pathogen virus

Similar to 2009 pandemic H1N1 virus.

Virus of avian origin but cultured from a man.

#### Real-time RT-PCR influenza assay

Viral RNA was isolated from 140 µl of each swab specimen and processed using the Qiagen: QIAamp Viral RNA Mini Kit (Qiagen Inc., Valencia, California) following a mini-spin protocol. Contaminants were washed away by two wash buffers and the RNA eluted in 50 µl of elution buffer. Specimens were screened for the presence of influenza A viral RNA using the CDC’s Human Influenza Virus Real-Time RT-PCR Detection and Characterization Panel [8]. This assay is a real-time RT-PCR (rRT-PCR) diagnostic for detection and characterization of influenza A virus. The primer and dual labeled hydrolysis probes in this system are capable of universal detection of influenza A virus while subtyping primer and probe sets are designed to specifically detect contemporary human A/H1, human 2009 pandemic H1N1, human A/H3, and avian A/H5 (Asian lineage) influenza viruses. Each extraction run included a mock extraction control to provide a secondary negative control to validate the extraction procedure and reagent integrity. The human RNase P gene primer set was used as an internal positive control for human RNA in each sample. Specimens that were rRT-PCR positive for generic influenza type A were further evaluated with a rRT-PCR procedure specific for human H1, H3, and H5, as well as 2009 pandemic H1. Swab samples positive and suspected positive for influenza A, but unable to be subtyped, were cultured in Madin-Darby canine kidney (MDCK) cells and passaged twice in an attempt to amplify the virus for further studies.

#### Hemagglutination Inhibition (HI) assay

A WHO-recommended HI assay [9] was utilized to test for serum antibodies against human influenza A viruses. Influenza virus strains were grown in MDCK cells or fertilized eggs. Sera were pre-treated with receptor destroying enzyme and hemabsorbed with either guinea pig or turkey erythrocytes. Titer results were reported as the reciprocal of the highest dilution of serum that inhibited virus-induced hemaglutination of a 0.65% (guinea pig) or 0.50% (turkey) solution of erythrocytes as previously established [10]. Viral antigens and control antisera for HI assays were obtained from acknowledged collaborators or from Biodefense and Emerging Infections (BEI) Research Resources Repository or through the Influenza Reagent Resource (IRR) program of the US CDC.

#### Microneutralization (MN) assay

A WHO-recommended MN assay adapted from that reported by Rowe [11], [12], [13] was used to detect human antibodies against equine and avian viruses. The viruses were grown in MDCK cells or fertilized eggs. Sera were first screened at a dilution of 1∶10. Positive specimens were then titered out in duplicate by examining 2-fold serial dilutions from 1∶10 to 1∶1280 in virus diluent [85.8% minimum essential medium (Invitrogen, Carlsbad, CA), 0.56% BSA, 25 mM HEPES buffer (Invitrogen), 100 mg/l streptomycin (Invitrogen), and 100,000 units/l penicillin (Invitrogen)]. Virus neutralization was then performed by adding 100 TCID_50_ of virus to the sera. The Reed Muench method was used to determine the TCID_50_/100 µL. [14] MDCK cells in log phase growth were adjusted to 2.0×10^5^ cells/mL with virus diluent. One hundred microliters of this suspension of cells were added to each well and the plates were then incubated at 37°C with 5% CO_2_ for 24 hours. Plates were washed twice with PBS, fixed with cold 80% acetone, and incubated at room temperature for 10 minutes. Influenza on the fixed monolayers was then quantified by influenza A nucleoprotein-specific indirect ELISA. The plates were washed with phosphate buffered saline containing 0.05% Tween 20 between each antibody addition after one hour incubation at room temperature. Following the final wash, 0.1 ml of 3,3′,5,5′- tetramethylbenzidine (TMB) (KPL 50-76-03) (Kirkegaard & Perry Laboratories Inc., Gaithersburg, Maryland) was added and incubated at room temperature for 10 mins. Color development will be stopped by the addition of 0.1 ml of 1N sulfuric acid. The optical density of the plates was read at 450 nm. The ELISA endpoint titer was expressed as the reciprocal of the highest dilution of serum with optical density (OD) less than X, where X = [(average OD of virus control wells)+(average OD of cell control wells)]/2. Test cells with an OD>2 times the cell control OD mean were considered positive for virus growth. The back titer was run in duplicate and only accepted when both replicates had matching results. Influenza viruses were obtained from acknowledged collaborators or from Biodefense and Emerging Infections (BEI) Research Resources Repository or through the Influenza Reagent Resource (IRR) program of the US CDC.

### Statistical Methods

Study outcomes included evidence of previous or acute influenza A virus infections. Acute influenza infection was defined as either a) isolation of influenza virus from a respiratory specimen obtained when a patient had an influenza-like illness, b) rRT-PCR evidence of influenza from such specimens, or c) a fourfold or greater rise in antibody titer against an influenza virus across annual follow-up sera. Because serologic responses to zoonotic influenza infections can rapidly wane [15], as we have reported previously [7], [16], [17], [18], we chose a low threshold of antibody titer (≥1∶10) as evidence of previous infection with an AIV or EIV strain. Because we know that cross-reactions from previous infection with human influenza viruses might confound AIV or EIV serology, our plan was to control such potential confounding by adding human influenza virus reactivity covariates to the multivariate models when the bivariate statistical analyses suggested they were important outcome predictors. Initially we examined risk factors for bivariate associations with assay results. When data were sparse we employed an exact method. Analyses were performed by using SAS v9.3 (SAS Institute, Inc., Cary, NC, USA).

## Results

During the period January through June 2009, 439 adult Mongolian participants were enrolled from 4 geographical areas (Khovd, Tuv, and Dornogovi aimags, as well as the capital city of Ulaanbaatar) in this prospective study of zoonotic influenza infections [7]. Among the participants at enrollment, 385 reported exposures to animals; 81 reported no such exposures (controls), and 52.2% were male. The average age of the participants was 39 yrs and only 4% reported ever receiving an influenza vaccine. Among the animal exposed participants, 48 (11%) reported exposures to poultry, and 334 (76%) reported recent exposure to horses. Seventy-five (17%) individuals reported recent disease outbreaks among their horses or camels.

A total of 363 participants (83%) completed the 12-month annual follow-up and 351 participants (80%) completed the 24-month annual follow-up visit. Overall, 307 participants (70%) remained enrolled for the entire study duration by completing enrollment and both 12- and 24-month follow-up visits. One hundred ILI investigations were conducted among 97 cohort subjects (3 subjects experienced 2 unique ILI events).

### Molecular Assay Results

Real-time RT-PCR analyses were performed on nasopharyngeal and throat swabs collected during ILI episodes. Thirty-six (36%) of the 100 symptomatic ILI cases had rRT-PCR evidence of influenza A inflection. Five (14%) of the 36 positives were identified as human H1N1 influenza virus. However, efforts to subtype the remaining 31 influenza A-positive specimens by rRT-PCR were unsuccessful, and none of the specimens yielded amplified virus after two passages in MDCK cells. Breaks in the cold chain were suspected as a cause of the nonviability of influenza viruses.

### Serologic Assay Results

Compared to serological results of enrollment sera [7], there was more AIV and EIV seroreactivity among 12- and 24-month follow-up sera (Table 2). Eight participants had elevated antibody titers against A/Hong Kong/1073/1999(H9N2) at either 12 months (4 subjects) or 24 months (4 subjects) (Table 3). Three subjects had ≥4-fold increases in antibody titers but did not report an ILI during the follow-up period, suggesting subclinical illnesses. Three participants had elevated antibody titers against A/Teal/Hong Kong/w312/97(H6N1) at 12 months (n = 1) or 24 months (n = 2); all had ≥4-fold increases in antibody titers between annual follow-up visits. Lastly, one participant had an elevated titer (1∶10) against A/Migratory duck/Hong Kong/MPD268/2007(H10N4) at 24 months. This subject experienced an ILI the month prior to her 24 month visit, although the respiratory swabs were negative for influenza A virus. Self-reporting exposure to wild birds or poultry was not associated with elevated titers against any of these viruses.

**Table 2:** Distribution of antibody titers against equine and avian influenza viruses among Mongolian participants, 2008–2011.

Titer	A/Equine/Mongolia/01/2008(H3N8)			A/Teal/Hong Kong/w312/97(H6N1)			A/Hong Kong/1073/1999(H9N2)			A/Migratory duck/Hong Kong/MPD268/2007(H10N4)		
	0 mo	12 mo	24 mo	0 mo	12 mo	24 mo	0 mo	12 mo	24 mo	0 mo	12 mo	24 mo
**<1∶10**	435	348	339	438	362	349	435	359	347	438	363	349
**1∶10**	3	11	5	1		1	3	3	2			1
**1∶20**	1	4	3		1	1		1	1			
**1∶40**			4						1			

0 mo = enrollment; 12 mo = 12 month annual follow-up; 24 mo = 24 month annual follow-up.

**Table 3:** Study participants with detectable microneutralization assays titers against A/Equine/Mongolia/01/2008(H3N8) or A/Hong Kong/1073/1999(H9N2), with seroconversions.

ID	Sex	Age(yrs)	H3N8(enroll)	H3N8(12 mo)	H3N8(24 mo)	H9N2(enroll)	H9N2(12 mo)	H9N2(24 mo)	Horse exposed during12 months before4-fold rise in titer	Poultry exposed during12 months before4-fold rise in titer
110	Male	45	<1∶10	1∶20*	1∶10				Yes	
308	Female	47	<1∶10	1∶20*	<1∶10				No	
360	Male	35	<1∶10	1∶20*	<1∶10				No	
302	Female	24	<1∶10	1∶20*	missing				No	
224	Female	38	<1∶10	1∶10	1∶40*				Yes	
270	Female	54	<1∶10	1∶10	1∶40*				Yes	
193	Female	52	<1∶10	1∶10	1∶20				Yes	
123	Male	44	<1∶10	1∶10	1∶10				Yes	
418	Male	28	<1∶10	1∶10	1∶10				No	
119	Male	48	<1∶10	1∶10	<1∶10				No	
319	Male	49	<1∶10	1∶10	<1∶10				No	
403	Male	35	<1∶10	1∶10	<1∶10				No	
100	Male	43	<1∶10	1∶10	missing				Yes	
102	Male	58	<1∶10	1∶10	missing				Yes	
362	Male	20	<1∶10	1∶10	missing				No	
297	Male	45	<1∶10	<1∶10	1∶40*				No	
298	Male	19	<1∶10	<1∶10	1∶40*				No	
186	Male	31	<1∶10	<1∶10	1∶20*				Yes	
182	Male	47	1∶10	<1∶10	1∶10				Yes	
290	Male	33	<1∶10	<1∶10	1∶10				No	
301	Male	40	<1∶10	missing	1∶20				Yes	
89	Male	57				<1∶10	1∶20*	<1∶10		No
68	Female	53				<1∶10	1∶10	<1∶10		No
222	Female	56				<1∶10	1∶10	<1∶10		No
240	Male	47				<1∶10	1∶10	<1∶10		No
221	Male	65				<1∶10	<1∶10	1∶40*		No
239	Male	55				<1∶10	<1∶10	1∶20*		No
338	Male	23				1∶10	missing	1∶10		Yes
337	Female	51				<1∶10	missing	1∶10		Yes

enroll = enrollment; 12 mo = 12 month annual follow-up; 24 mo = 24 month annual follow-up;

= 4-fold rise in titer between 2 timer periods;

Fifteen participants (4.1%) had elevated antibody titers against A/Equine/Mongolia/01/2008(H3N8) at the 12-month visit, and 12 participants (3.4%) had elevated titers at 24 months (Table 3). Six participants were seropositive at both follow-up visits; one participant with an elevated H3N8 titer at 24 months was also seropositive at enrollment. Eleven subjects had evidence of subclinical or mild EIV infections identified through ≥4-fold increases in antibody titers between annual follow-up visits, yet did not report an ILI event during the respective follow-up period. Among the 27 EIV seropositive participants, exposure to horses and/or camels was not associated with elevated titers against EIV. Elevated titers against the 3 human influenza viruses (H1N1, H3N2, and pandemic H1N1) were also not significantly associated with elevated EIV titers.

In contrast, there was considerable seroreactivity against human influenza viruses during the 2 yrs of follow-up. At 12 months, 164 participants (45.3%) had elevated antibody titers against A/Brisbane/59/2007(H1N1); 97 participants (26.7%) had elevated antibody titers against A/Brisbane/10/2007(H3N2); and 22 (6.7%) had elevated antibody titers against A/Mexico/4108/2009(H1N1). At 24 months, 52 participants (14.8%) had elevated antibody titers against A/Brisbane/59/2007(H1N1); 322 participants (91.7%) had elevated antibody titers against A/Brisbane/10/2007(H3N2); and 87 (26.4%) had elevated antibody titers against A/Mexico/4108/2009(H1N1).

## Discussion

While the occurrence of seroreactivity against AIV and EIV among the study population was higher during the 12 and 24 month follow-up periods compared to their seroreactivity upon enrollment, specific risk factor associations could not be identified. It seems likely that AIV and EIV serologic findings were confounded by cross-reacting antibodies against human influenza viruses. Evidence for this includes the relatively low magnitudes of the microneutralization titers against AIV and EIV, our inability to associate these elevations or 4-fold titer increases with animal exposures, a high prevalence of elevated antibodies against human seasonal H1N1 and H3N2 viruses, and the temporal association between outbreaks of human H1N1 [19] and H3N2 [20] infections during the follow-up period.

This study has a number of limitations. Our three rural specimen collection sites did not have −80°C freezers and sites were located at varying distances from the central laboratory where testing of study specimens took place. These factors likely caused disruptions in the viral cold chain, which could have hindered our ability to detect and isolate viruses from study specimens, though the 5 H1N1 flu A positive samples were well distributed across the 3 aimags. While we did not find strong evidence of AIV or EIV infection among our study subjects who were all adults, it could be true (as was anecdotally reported by Mongolian herders) that children being more immunologically naïve are more likely to suffer zoonotic influenza infections. Unfortunately, we did not target children in this research study and were not able to examine such anecdotal observations.

It seems prudent to continue to conduct zoonotic influenza studies among Mongolia’s people, wildlife, and domestic animals. Mongolia has experienced multiple incursions of highly pathogenic avian influenza strains, and multiple large epizootics of equine influenza. Mongolians are some of the world’s last nomadic people and they live in close proximity to their animals. However, as AIV and EIV infections in man are likely rare events in Mongolia, it would take much larger cohort studies to estimate incidence and to identify risk factors for such infections. Due to weak public health infrastructure in Mongolia, problems with maintaining the cold chain, and the cost and technical difficulty of conducting the tedious serological assays, such large studies seem cost-prohibitive at least for the near future.
